# Podocalyxin promotes an impermeable epithelium and inhibits pro-implantation factors to negatively regulate endometrial receptivity

**DOI:** 10.1038/s41598-021-03425-2

**Published:** 2021-12-14

**Authors:** Sophea Heng, Nirukshi Samarajeewa, Yao Wang, Sarah G. Paule, James Breen, Guiying Nie

**Affiliations:** 1 Implantation and Pregnancy Research Laboratory, School of Health and Biomedical Sciences, RMIT University, Bundoora West Campus, Melbourne, VIC 3083 Australia; 2 Hudson Institute of Medical Research, Clayton, VIC Australia; 3 The Robinson Research Institute, University of Adelaide, Adelaide, SA Australia; 4 University of Adelaide Bioinformatics Hub, School of Biological Sciences, University of Adelaide, Adelaide, SA Australia; 5 South Australian Health & Medical Research Institute, Adelaide, SA Australia

**Keywords:** Cell biology, Molecular biology

## Abstract

Embryo implantation is a key step in establishing pregnancy and a major limiting factor in IVF. Implantation requires a receptive endometrium but the mechanisms governing receptivity are not well understood. We have recently discovered that podocalyxin (PCX or PODXL) is a key negative regulator of human endometrial receptivity. PCX is expressed in all endometrial epithelial cells in the non-receptive endometrium but selectively down-regulated in the luminal epithelium at receptivity. We have further demonstrated that this down-regulation is essential for implantation because PCX inhibits embryo attachment and penetration. However, how PCX confers this role is unknown. In this study, through RNAseq analysis of Ishikawa cell line stably overexpressing PCX, we discovered that PCX suppresses expression of genes controlling cell adhesion and communication, but increases those governing epithelial barrier functions, especially the adherens and tight junctions. Moreover, PCX suppresses multiple factors such as LIF and signaling pathways including Wnt and calcium signaling that support receptivity but stimulates anti-implantation genes such as LEFTY2. Functional studies confirmed that PCX promotes epithelial barrier functions by increasing key epithelial junction proteins such as E-cadherin and claudin 4. PCX thus promotes an anti-adhesive and impermeable epithelium while impedes pro-implantation factors to negatively control endometrial receptivity for implantation.

## Introduction

Embryo implantation is a key step in establishing pregnancy. It requires coordinated interactions between a well-developed embryo and a receptive endometrium (the inner lining of the uterus)^1–4^. Implantation failure is a major cause of infertility and a key obstacle in IVF to treat infertility^5^. Innovations in embryo culture and selection have significantly improved IVF outcomes, yet the average live birth rate remains to be further increased^6–8^, and the endometrium is considered a major target to improve implantation rates^9–11^.

The process of human implantation differs considerably from other species^12–15^. Human implantation requires the embryo to attach to the endometrial luminal epithelium, traverse the epithelial layer, penetrate the basement membrane underneath, and imbed itself within the stromal compartment^3,14^. The luminal epithelium then reseals over the implantation site, completely encapsulating the embryo within the tissue^14^. Therefore, the luminal epithelium, which lines the uterine cavity and is the initial point of contact by an embryo for implantation, is highly critical for endometrial receptivity.

Despite years of research and numerous transcriptome studies of endometrial tissue^16^, mechanisms that regulate endometrial receptivity remain to be fully elucidated. Nevertheless, a widely held view is that endometrial receptivity requires up-regulation of adhesion-promoting molecules and cytokines^17–19^ to facilitate embryo attachment, yet the details are not well understood. However, our recent studies suggest a rather contrasting mechanism: the human endometrial epithelium intrinsically expresses an anti-implantation factor which prevents receptivity, and the luminal epithelium must reduce this negative regulator to switch the endometrial surface from a non-receptive to an implantation-permitting state^20,21^. This negative regulator is glycoprotein podocalyxin (PCX, also known as PODXL, PCLP1, gp135, MEP21, and thrombomucin).

In the non-receptive human endometrium, PCX is strongly expressed on the apical surface of all luminal and glandular epithelial cells as well as vascular endothelial cells. However, when the endometrium becomes receptive for implantation in the mid-secretory phase of the menstrual cycle, luminal PCX is selectively and specifically down-regulated to below detection^20^. We have functionally validated that PCX indeed negatively controls endometrial receptivity^20^. When PCX is overexpressed in Ishikawa cells, a commonly used receptive human endometrial epithelial cell line, the cellular characteristics profoundly converted into a non-receptive state. Importantly, the PCX-overexpressing (PCX-OE) cells significantly inhibit the attachment as well as the penetration of embryo mimics^20^. These cells likewise inhibit the implantation of actual human embryos in in vitro models^21^. Moreover, in IVF patients inadequate down-regulation of PCX in the endometrial luminal epithelium around the time of embryo transfer is significantly associated with implantation failure^21^. These results highlight the clinical significance of endometrial PCX regulation in fertility treatment. However, it remains to be comprehended how PCX functions as a negative regulator of endometrial epithelial receptivity.

PCX is a large transmembrane protein belonging to the CD34 family of sialomucins^22^. It is primarily expressed in podocytes (specialized kidney epithelial cells), vascular endothelial cells, mesothelial cells, hematopoietic progenitor cells, epithelial cells and many carcinomas^23–25^. In podocytes, PCX acts as an anti-adhesive protein and plays an essential role in the formation and maintenance of podocyte foot processes^25^. PCX is reported to interact with adaptor proteins that are implicated in protein trafficking, ion transport, signaling^26–28^ and actin binding^29,30^. Recent studies in endothelial cells show that PCX plays a key role in maintaining the blood–brain barrier function especially during acute inflammation^31^. Knockdown of PCX in human umbilical vein endothelial cells leads to disorganization of actin filaments, impairment of cell to cell interactions, and mis-localization of adherens junctions^31^. However, it is unknown whether PCX also regulates the barrier functions of epithelial cells.

In this study, we investigated how PCX regulates epithelial cell gene expression and cellular properties that particularly pertinent to endometrial receptivity. We leveraged our previously established Ishikawa cell line that stably overexpresses PCX and conducted RNA sequencing (RNAseq) analysis. We then validated significant findings by functional studies.

## Results

### PCX reduces expression of genes controlling cell adhesion and communication but increases those governing epithelial barrier functions

**Figure 1:**
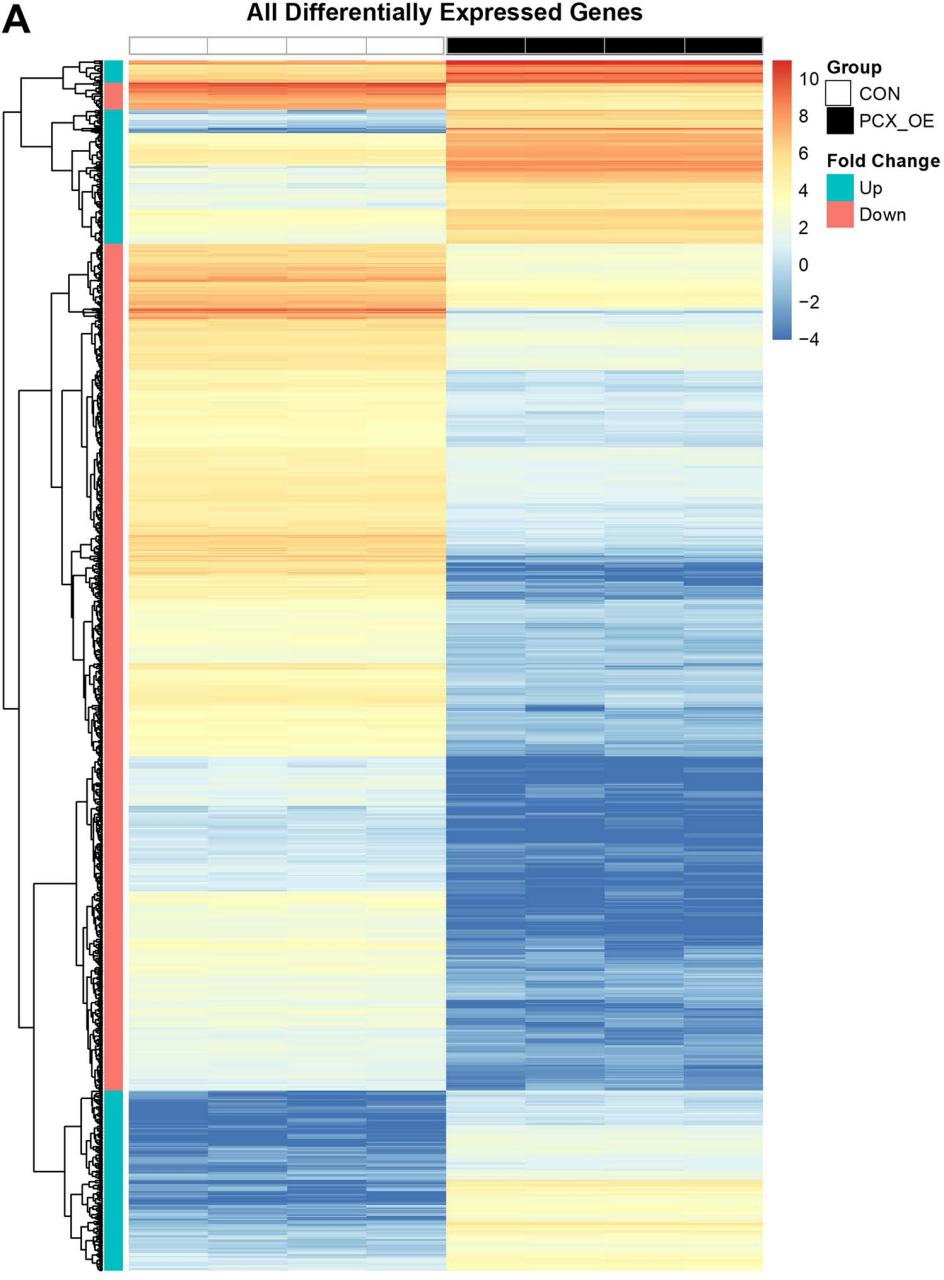

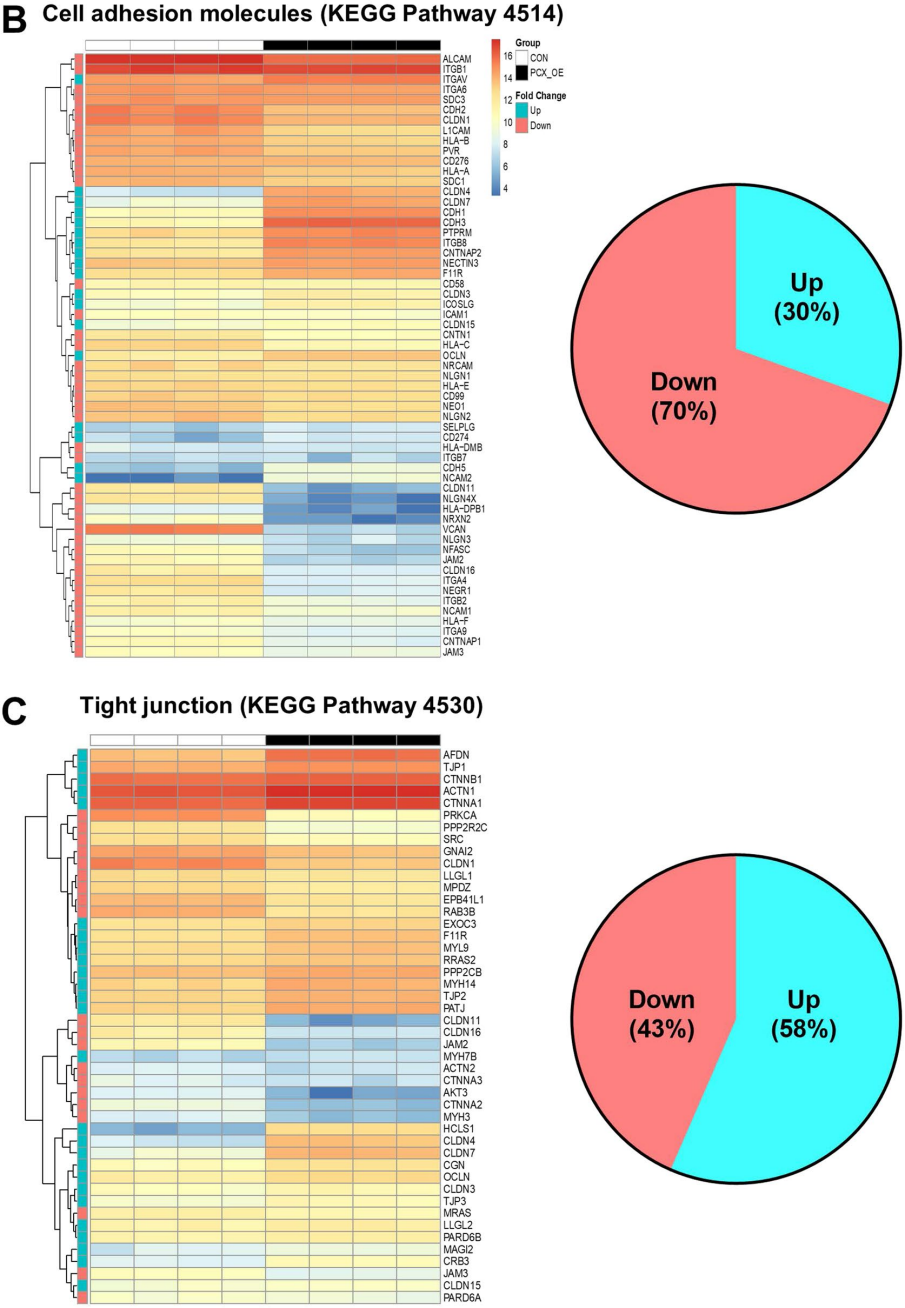

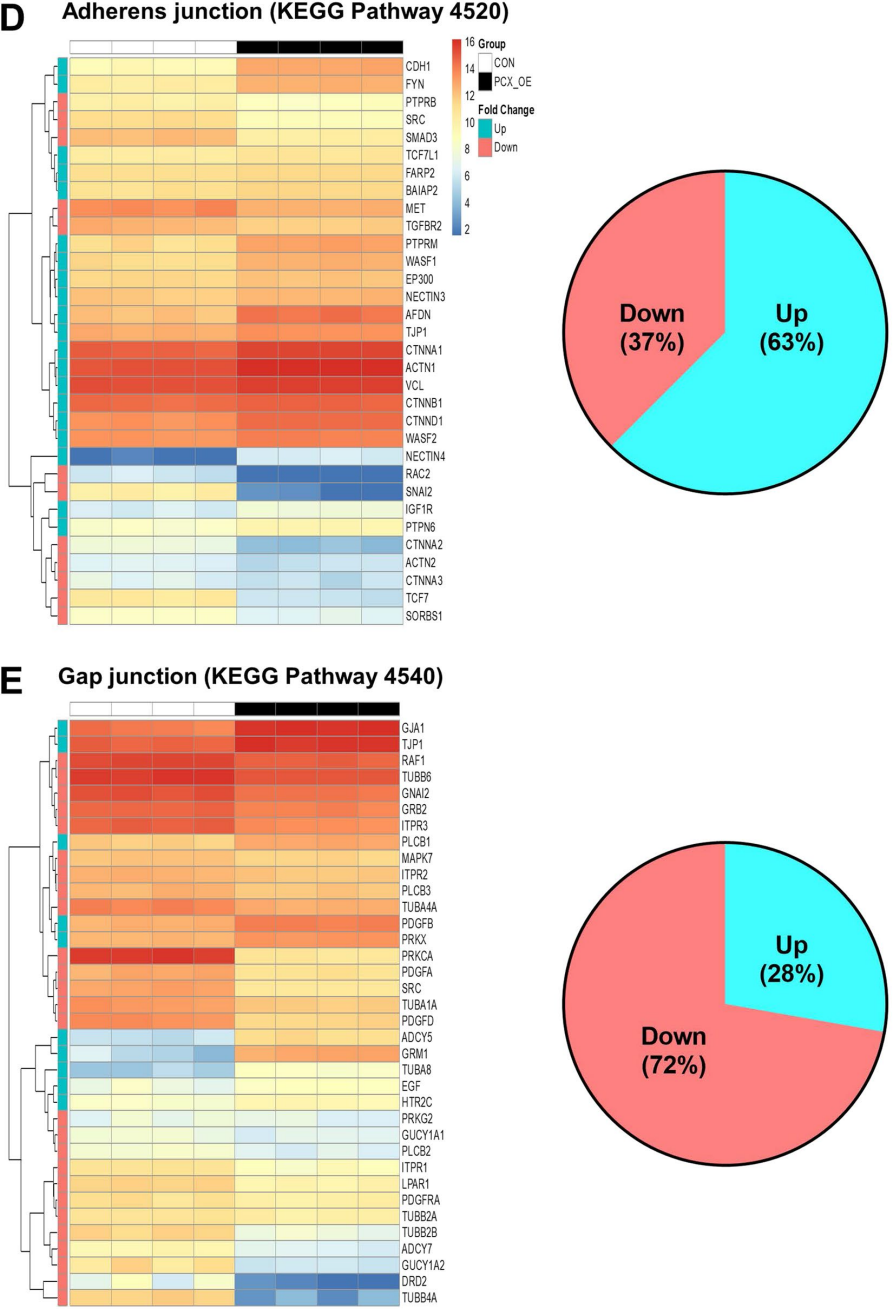
RNAseq analysis of control and PCX-overexpressing Ishikawa cells. (**A**) Heat-map of all differentially expressed genes between control (CON) and PCX-overexpressing (PCX-OE) Ishikawa cells. Normalized gene expression values (Transcripts Per Million, TPM) are outlined in each heatmap. (**B–E**) Heat-map of differentially expressed genes associated with KEGG gene pathways (https://www.genome.jp/kegg/pathway.html) for cell adhesion and junction. Each pathway is labelled with the KEGG pathway database ID (e.g. “Cell adhesion molecules” has the KEGG pathway ID 4514) (**B**) Cell adhesion molecules. (**C**) Tight junction. (**D**) Adherens junction. (**E**) Gap junction. For each, proportions of up- (Up, in blue) and down-regulated (Down, in red) genes are also shown by a pie chart.

To understand how PCX negatively regulates endometrial receptivity, we compared gene expression of control (receptive) and PCX-OE (non-receptive) Ishikawa cells by RNAseq. A total of 15,103 genes were expressed in both cell types after filtering for low counts, however, unsupervised clustering analysis clearly separated the control and PCX-OE cells into two distinct groups (Supplementary Figure S1). Based on the criteria of false discovery rate < 0.01 and log (fold change) > 2 or < − 2, a total of 940 genes were found to be differentially expressed between the two groups (Fig. 1A). Of these, 659 were down-regulated, whereas 281 were up-regulated, in PCX-OE cells compared to the control (Fig. 1A).

Since cell adhesion and epithelial junctions are particularly relevant to embryo attachment and penetration for implantation, we closely examined the transcriptional changes associated with these cellular features (Fig. 1B–E). For cell adhesion related molecules (Fig. 1B), 59 genes were differentially expressed between the two groups, however the majority (70%) were down-regulated in PCX-OE cells compared to the control (Fig. 1B). For tight junction genes, 46 were differentially expressed, but they were preferentially (58%) up-regulated in PCX-OE cells (Fig. 1C). A similar trend was observed for the adherens junction, for which 32 genes differed in their expression, and 63% of them were up-regulated in PCX-OE than control cells (Fig. 1D). However, the gap junction showed a contrasting pattern, and among the 36 differentially expressed genes, 72% were down-regulated in PCX-OE than control cells (Fig. 1E). These data suggest that PCX reduces expression of genes controlling cell adhesion and cell–cell communication, but increases those governing epithelial barrier functions.

### PCX down-regulates multiple signaling pathways that support endometrial receptivity

**Figure 2:**
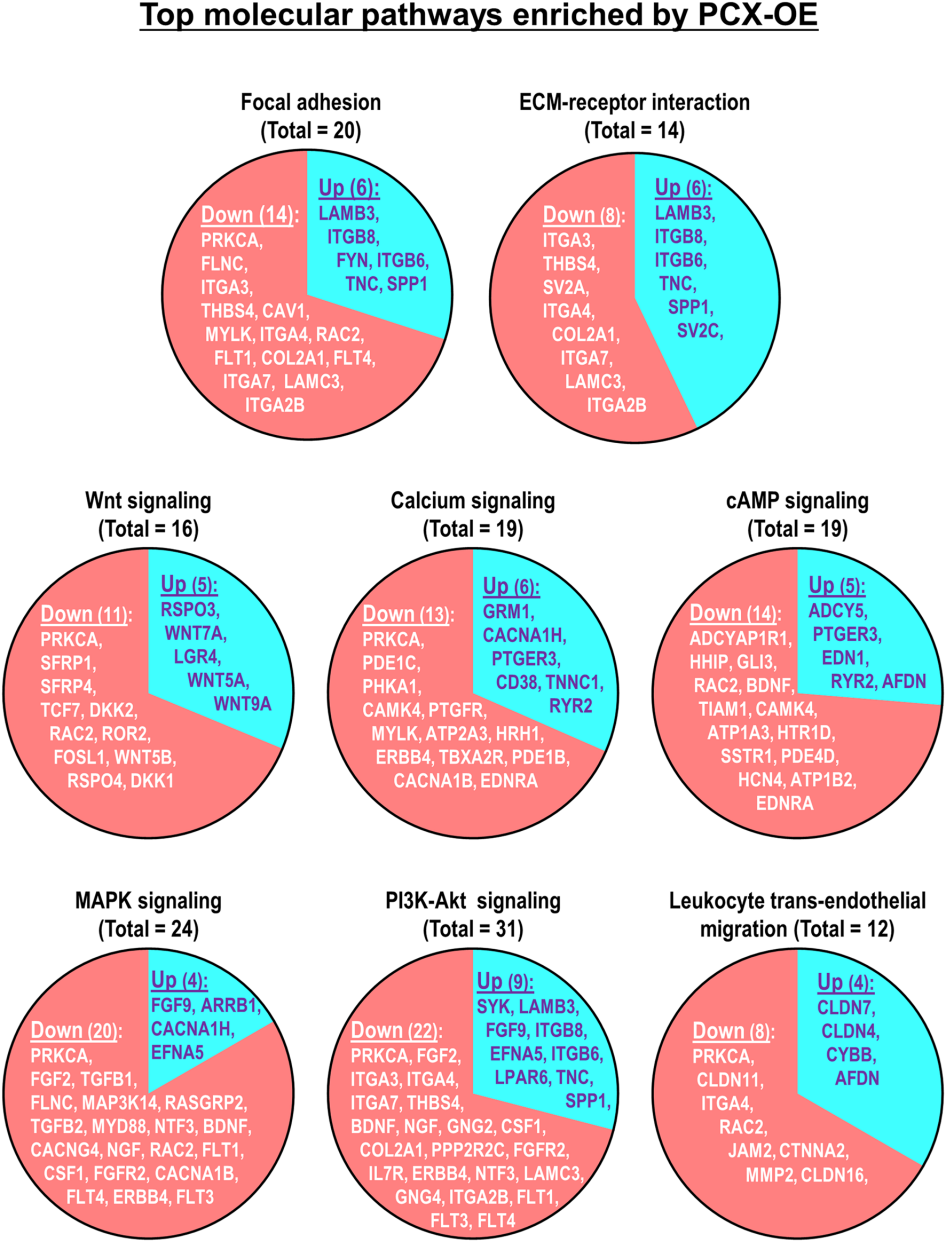
Top molecular pathways enriched by PCX-overexpression. Each pathway is presented by a pie chart, within which the total number and names of genes that are up-regulated (Up, in blue) and down-regulated (Down, in red) in PCX-overexpressing (PCX-OE) than control (CON) Ishikawa cells are shown.

We next performed pathway enrichment analysis on all differentially expressed genes, which identified 20 KEGG pathways (Table 1). Strikingly, all these enriched pathways were predominantly down-regulated in PCX-OE cells compared to the control (Table 1). Top 9 of these pathways are particularly relevant to endometrial receptivity and embryo implantation, which included focal adhesion, ECM-receptor interaction, signaling of Wnt, calcium, cAMP, MAPK and PI3K-Akt, as well as leukocyte trans-endothelial migration (Table 1, Fig. 2), in addition to cell adhesion (Table 1, discussed earlier). These are all known to positively influence endometrial receptivity^32–39^. For all these pathways, more genes were down- than up-regulated in PCX-OE cells compared to the control (Table 1, Fig. 2). These data suggest that PCX induces down-regulation of genes of multiple signaling pathways that support endometrial receptivity.

**Table 1:** Selected KEGG gene pathways enriched by POD-OE in Ishikawa cells using limma.

Enriched pathways	Number of genes altered			Specific genes involved	
	Total	Up	Down	Up-regulated	Down-regulated
Cell adhesion molecules (CAMs)	19	6	13	CDH3, CDH1, CLDN7, CLDN4, ITGB8, NCAM2,	VCAN, NLGN4X, CLDN11, NEGR1, ITGA4, NFASC, JAM2, LRRC4C, HLA-C, NRXN2, HLA-DPB1, CNTNAP1, CLDN16
ECM-receptor interaction	14	6	8	LAMB3, ITGB8, ITGB6, TNC, SPP1, SV2C,	ITGA3, THBS4, SV2A, ITGA4, COL2A1, ITGA7, LAMC3, ITGA2B
Focal adhesion	20	6	14	LAMB3, ITGB8, FYN, ITGB6, TNC, SPP1	PRKCA, FLNC, ITGA3, THBS4, CAV1, MYLK, ITGA4, RAC2, FLT1, COL2A1, FLT4, ITGA7, LAMC3, ITGA2B
Wnt signaling pathway	16	5	11	RSPO3, WNT7A, LGR4, WNT5A, WNT9A,	PRKCA, SFRP1, SFRP4, TCF7, DKK2, RAC2, ROR2, FOSL1, WNT5B, RSPO4, DKK1
Calcium signaling pathway	19	6	13	GRM1, PTGER3, CACNA1H, CD38, TNNC1, RYR2	PRKCA, PDE1C, PHKA1, CAMK4, PTGFR, MYLK, ATP2A3, TBXA2R, PDE1B, HRH1, CACNA1B, ERBB4, EDNRA
cAMP signaling pathway	19	5	14	ADCY5, PTGER3, EDN1, RYR2, AFDN	ADCYAP1R1, HHIP, GLI3, CAMK4, BDNF, TIAM1, RAC2, ATP1A3, HTR1D, SSTR1, PDE4D, HCN4, ATP1B2, EDNRA
MAPK signaling pathway	24	4	20	FGF9, ARRB1, EFNA5, CACNA1H	PRKCA, FGF2, TGFB1, FLNC, MAP3K14, TGFB2, MYD88, NTF3, BDNF, NGF, RAC2, FLT1, CSF1, FGFR2, CACNA1B, RASGRP2, FLT4, ERBB4, FLT3, CACNG4
PI3K-Akt signaling pathway	31	9	22	SYK , LAMB3, FGF9, ITGB8, EFNA5, ITGB6, TNC, SPP1, LPAR6	PRKCA, FGF2, ITGA3, NTF3, THBS4, BDNF, NGF, ITGA4, GNG2, FLT1, CSF1, COL2A1, PPP2R2C, FGFR2, GNG4, FLT4, ERBB4, ITGA7, LAMC3, IL7R, FLT3, ITGA2B
Mucin type O-glycan biosynthesis	9	4	5	GALNT3, GCNT3, ST6GALNAC1, GALNT13	GALNT14, GALNT18, GALNT6, GALNT16, GALNT5
Leukocyte transendothelial migration	12	4	8	CLDN7, CLDN4, CYBB, AFDN,	PRKCA, CLDN11, ITGA4, RAC2, JAM2, CTNNA2, MMP2, CLDN16,
Hematopoietic cell lineage	11	2	9	IL1R2, CD38	ITGA3, MME, ITGA4, CSF1, HLA-DPB1, ANPEP, IL7R, FLT3, ITGA2B
Insulin secretion	10	3	7	ADCY5, RYR2, KCNN3	PRKCA, ADCYAP1R1, ABCC8, ATP1A3, KCNN1, RIMS2, ATP1B2
Pathways in cancer	41	10	31	CDH1, LAMB3, WNT7A, WNT5A, FGF9, ADCY5, PTGER3, EDN1, LPAR6, WNT9A	PRKCA, FGF2, STAT6, TGFB1, ITGA3, TGFB2, HHIP, GLI3, BMP4, AR, TCF7, GNG2, RAC2, NKX3-1, WNT5B, CASP7, STAT5A, GLI2, FGFR2, HEYL, GNG4, RASGRP2, FLT4, CTNNA2, MMP2, LAMC3, IL7R, FLT3, EDNRA, ITGA2B, RUNX1T1
Basal cell carcinoma	9	3	6	WNT7A, WNT5A, WNT9A	HHIP, GLI3, BMP4, TCF7, WNT5B, GLI2
Axon guidance	18	5	13	EPHA1, SEMA4A, WNT5A, EFNA5, FYN	PRKCA, SEMA5A, DPYSL2, SEMA3D, SEMA6B, NGEF, RAC2, EPHA4, WNT5B, LRRC4C, DPYSL5, TRPC6, PLXNA2
Dilated cardiomyopathy (DCM)	12	5	7	ADCY5, ITGB8, ITGB6, TNNC1, RYR2	TGFB1, ITGA3, TGFB2, ITGA4, ITGA7, CACNG4, ITGA2B
Hypertrophic cardiomyopathy (HCM)	13	5	8	ITGB8, EDN1, ITGB6, TNNC1, RYR2,	TGFB1, ITGA3, TGFB2, ITGA4, ACE, ITGA7, CACNG4, ITGA2B
Arrhythmogenic right ventricular cardiomyopathy (ARVC)	10	3	7	ITGB8, ITGB6, RYR2	ITGA3, TCF7, ITGA4, ITGA7, CTNNA2, CACNG4, ITGA2B
Histidine metabolism	5	0	5		ALDH3A1, MAOB, MAOA, AOC1, ALDH3B1
Morphine addiction	15	4	11	GABRP , ADCY5, ARRB1, KCNJ5	PRKCA ,PDE1C, GNG2, PDE1B, CACNA1B, GNG4, PDE4D, PDE7B, KCNJ3, GABRG2, GABRA2

Each enriched gene pathway set selected had an adjusted p-value of 0.01, with the number of genes altered in up and down directions detailed.

### PCX inhibits receptivity-promoting factors but up-regulates anti-implantation genes

**Figure 3:**
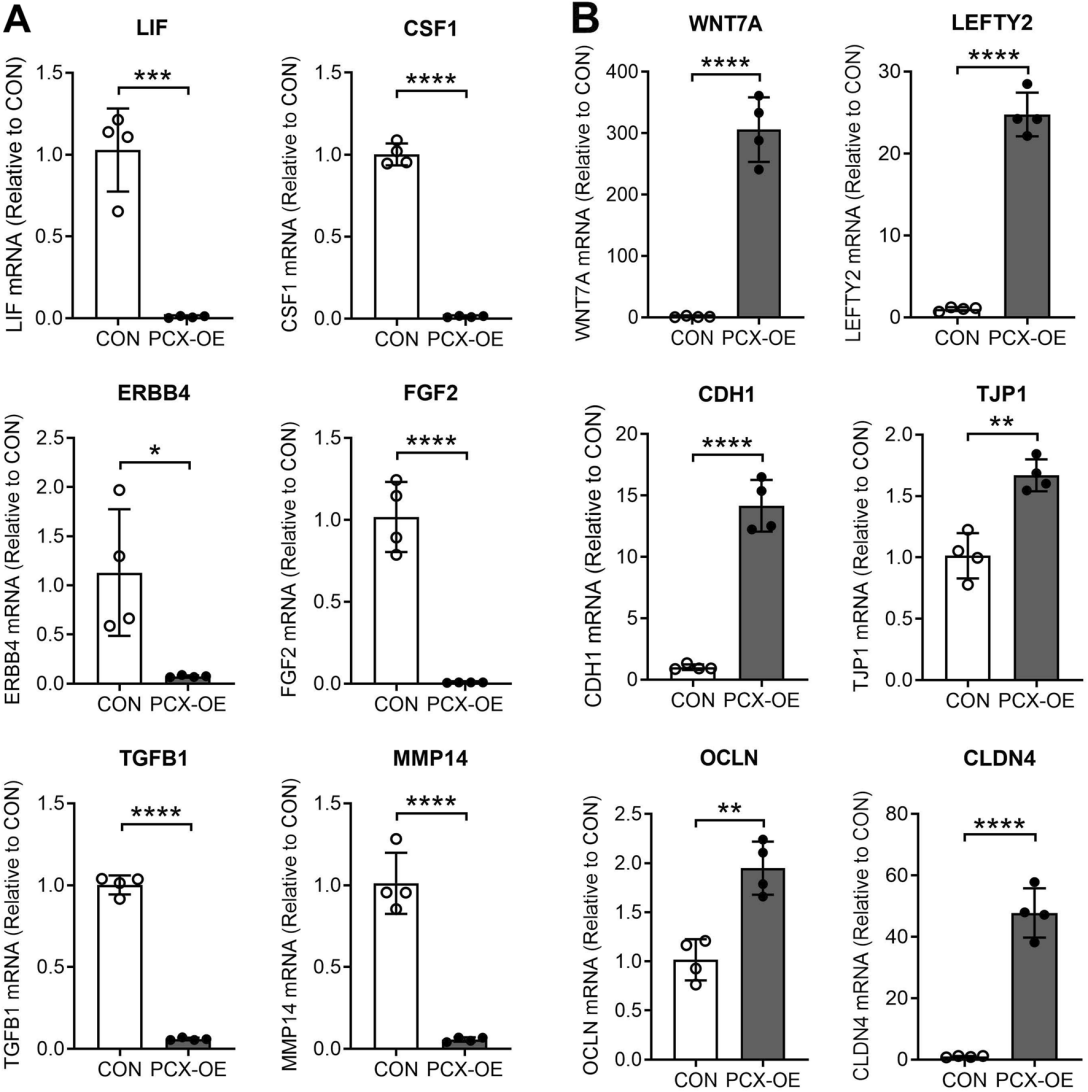
Real-time RT-PCR validation of differentially regulated genes between control and PCX-overexpressing Ishikawa cells. (**A**) Down-regulation of *LIF, CSF1*, *ERBB4*, *FGF2, TGFB1* and *MMP14* in PCX-overexpressing (PCX-OE) compared to control (CON) cells. (**B**) Up-regulation of *WNT7A*, *LEFTY2*, *CDH1, TJP1, CLDN4 * and *OCLN* in PCX-OE compared to CON cells. Data normalized to YWHAZ as a house keeping gene and expressed as fold change relative to CON (mean ± SD, n = 4). *P < 0.05, **P < 0.005, ***P < 0.0005, ****P < 0.0001.

We further inspected the RNAseq dataset for effect of PCX overexpression on specific genes that are known to be associated with endometrial receptivity and embryo implantation, and then validated their changes by real-time RT-PCR. A number of receptivity promoting factors, including *LIF* (interleukin 6 family cytokine), *CSF1* (colony stimulating factor 1), *ERBB4* (HER4), *FGF2* (fibroblast growth factor 2), *TGFB1* (TGF-beta-1), and the matrix metallopeptidases *MMP14* (MT1-MMP)^40–45^, were markedly suppressed in PCX-OE cells compared to the control (Fig. 3A).

In contrast, two endometrial factors that are linked to implantation failure, *WNT7A* (Wnt family member 7A) and *LEFTY2* (left–right determination factor 2)^46,47^, were markedly up-regulated in PCX-OE cells compared to the control (Fig. 3B). We also validated by RT-PCR the PCX-mediated increases in key tight and adherens junction candidates, and data for *CDH1* (E-cadherin), *TJP1* (ZO-1), *OCLN* (occludin) and *CLDN4* (claudin 4) are shown in Fig. 3B.

Collectively, these results suggest that PCX suppresses the expression of genes that are associated with the attainment of receptivity but stimulates those that are related to a non-receptive state.

### Immunofluorescence validation that PCX increases key epithelial junction proteins

**Figure 4:**
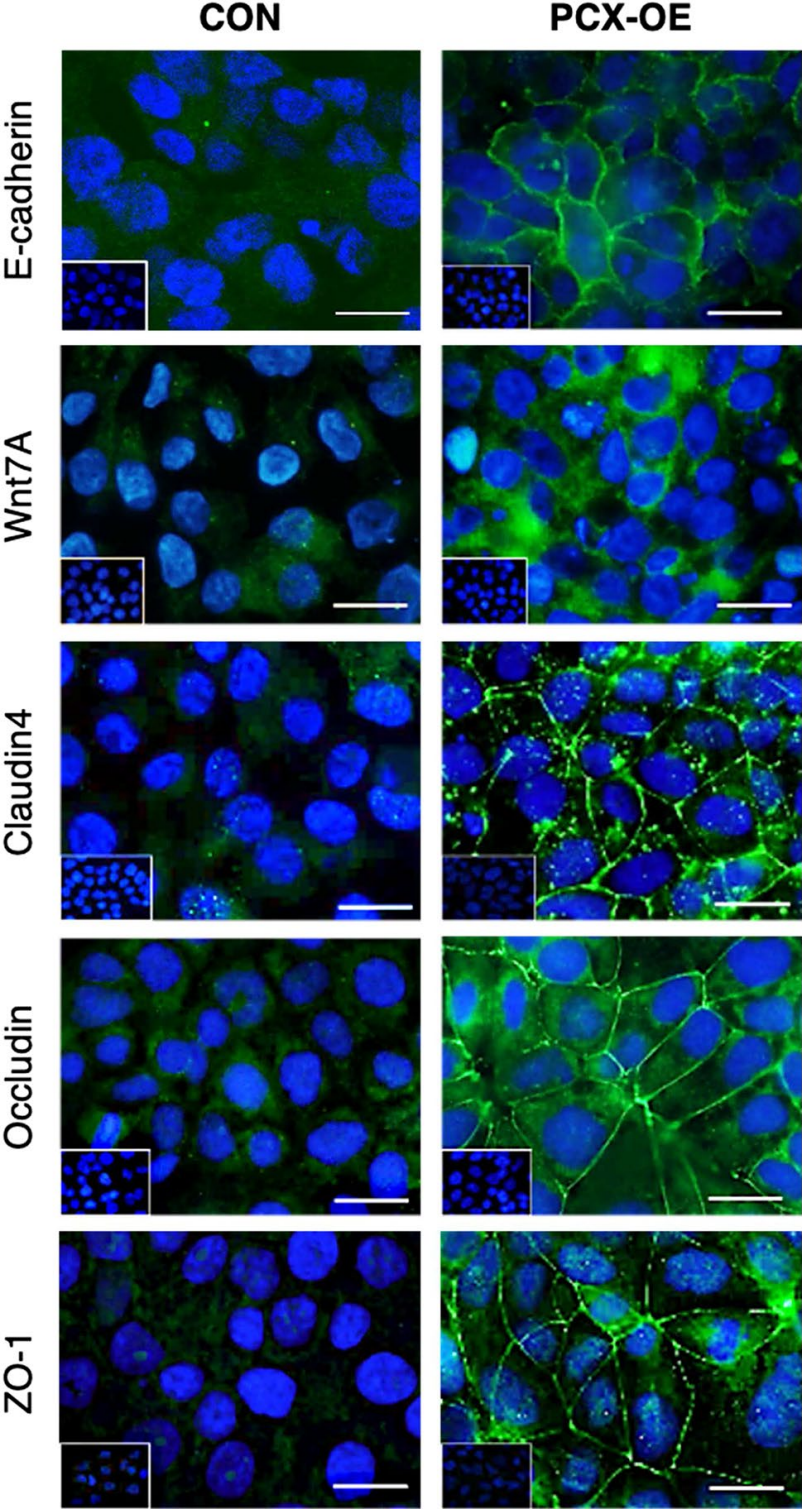
Immunofluorescence of key epithelial junction proteins. Analysis of E-cadherin and Wnt7A (adherens junction), claudin4 and occluding (tight junction), and ZO1 (cytoskeletal connection) in control (CON) and PCX-overexpressing (PCX-OE) cells. All candidates are in green and nuclei in blue. Inserts, negative controls. Scale bars, 20 µm.

Since a major functional feature of PCX-OE Ishikawa cells was inhibition of embryo invasion through the monolayer^20,21^, we further examined key epithelial junction proteins by immunofluorescence. Adherens junction proteins E-cadherin and Wnt7A were clearly increased in PCX-OE cells compared to control (Fig. 4), consistent with their mRNA being up-regulated by PCX-OE. Tight junction proteins claudin 4 and occludin showed a higher level in PCX-OE than control cells (Fig. 4), in concord with their mRNA changes. Cytoskeletal connector ZO-1 was likewise clearly intensified in PCX-OE cells (Fig. 4). All these proteins showed a much more prominent localization to cell junctions in PCX-OE than control cells (Fig. 4), demonstrating that PCX promotes the formation of tight and adherens junctions in the Ishikawa monolayer.

### Functional validation that PCX increases epithelial barrier functions

**Figure 5:**
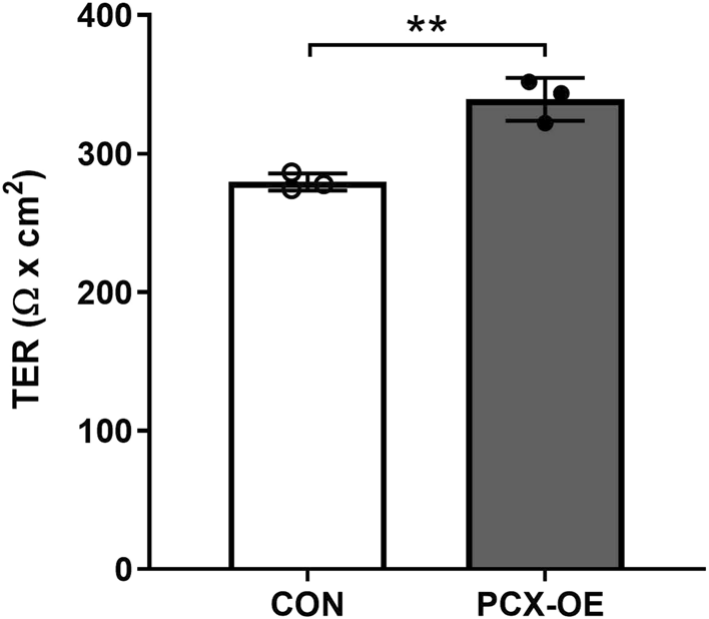
PCX increases trans-epithelial electrical resistance. Analysis of trans-epithelial electrical resistance (TER) in control (CON) and PCX-overexpressing (PCX-OE) Ishikawa cells. Data expressed as mean ± SD (n = 3). **P < 0.005.

**Figure 6:**
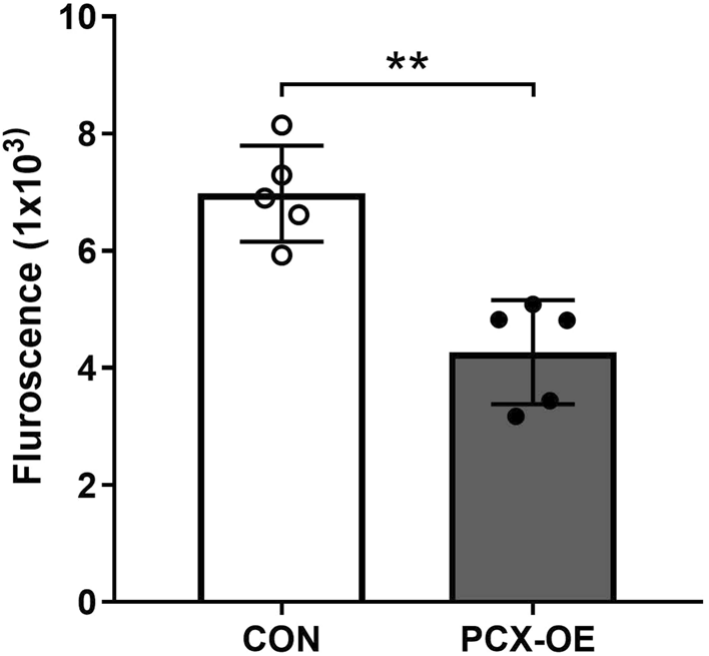
PCX decreases epithelial barrier permeability. Analysis of FITC-dextran flux from the top to the bottom monolayer of control (CON) and PCX-overexpressing (PCX-OE) cells. Data expressed as mean ± SD (n = 5). **P < 0.005.

To confirm that the above changes in junction genes and proteins shown in Figs. 3 and 4 confer epithelial barrier functions, we next measured the “tightness” of Ishikawa cell–cell connections using trans-epithelial electrical resistance (TER) across the monolayer as a biophysical measurement. TER was significantly increased in PCX-OE monolayer compared to the control (Fig. 5, **P = 0.0034), consistent with PCX enhancing the epithelial barrier integrity. To further confirm this observation, we next assessed epithelial barrier permeability, by measuring the capacity of FITC-labelled dextran (Mol wt 40 kDa) to permeate the Ishikawa monolayer. The flux of FITC-dextran through the monolayer from the top to the bottom was significantly lower in PCX-OE than control cell (Fig. 6, **P = 0.001), again in accordance with PCX promoting a tighter epithelial barrier. These findings validated that PCX increases epithelial barrier functions.

## Discussion

**Figure 7:**
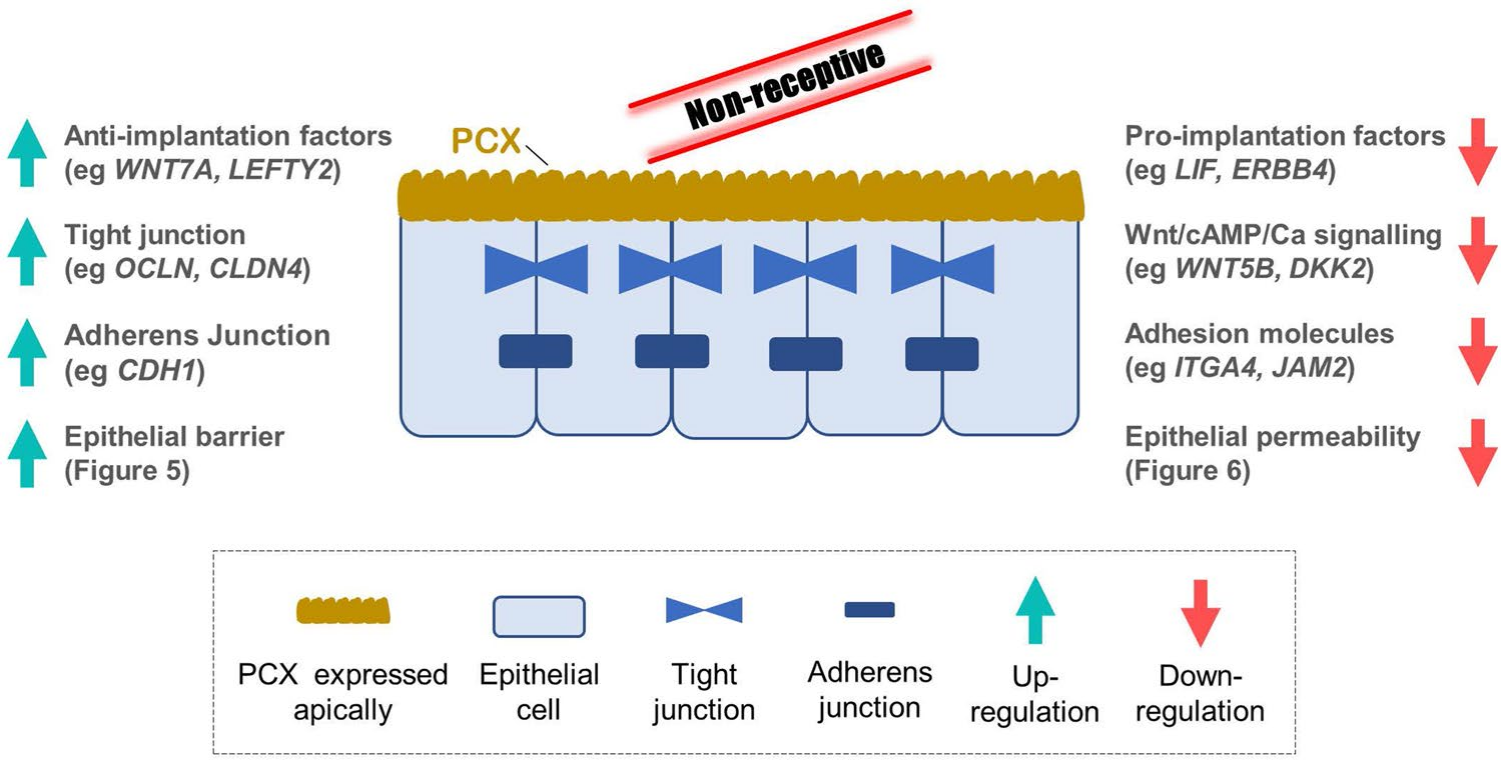
Proposed mechanisms of PCX function in negatively regulating endometrial receptivity. PCX acts as an epithelial sealant and anti-implantation factor, rendering the endometrial epithelium non-adhesive and impermeable for embryos to attach or traverse.

Successful embryo implantation requires a receptive endometrium. While previous research has provided important insights into endometrial remodeling for receptivity^5,15,16^, mechanisms governing endometrial surface receptivity is not well understood. We recently discovered that PCX plays a key role in negatively controlling human endometrial epithelial receptivity. In the current study we revealed the potential underling mechanisms that confer this function of PCX—it promotes factors that are anti-implantation and increases those that are supporting tight and adherens junctions, and at the same time PCX hinders pro-implantation factors/pathways and decreases adhesion molecules. We thus propose that PCX acts as an epithelial sealant and anti-implantation factor, rendering the endometrial epithelium non-adhesive and impermeable for embryos to attach to or transverse through. Figure 7 depicts these proposed mechanisms of PCX function in negatively controlling endometrial receptivity. These results provide important knowledge in the understanding of endometrial receptivity as well as epithelial biology in general.

Our data showed that PCX induces down-regulation of adhesion molecules in endometrial Ishikawa cells, these results are highly consistent with the anti-adhesive role of PCX previously identified in kidney podocytes^25^. However, our studies greatly expanded this narrative. Our RNAseq analysis revealed for the first time that PCX also influences the expression of hundreds of genes that are associated with epithelial junctions. In particular, PCX profoundly enhances gene expression that governs the tight and adherens junctions but at the same time preferentially reduces those dictate the gap junction. The end results would be an anti-adhesive and impermeable epithelium, which we experimentally validated by immunofluorescence and functional studies. These epithelial profiles are consistent with PCX preventing endometrial receptivity as we have previously reported^20,21^. These newly identified roles of PCX in promoting epithelial barrier functions are consistent with the findings recently reported in endothelial cells^31^. These barrier-protecting roles of PCX^20,21^ may shed new light on the necessity of its widespread expression in epithelial, endothelial and mesothelial cells throughout the human body. Our results thus have broader implications in the understanding of PCX in organs outside the endometrium.

In addition to being anti-adhesive and anti-permeability, PCX also dampens the expression of a number of pro-implantation factors but increases those that are known to inhibit implantation. LIF is a well-known receptivity promoting factor for embryo implantation^48^. Down-regulation of LIF is associated with unexplained infertility and recurrent pregnancy loss^49,50^. Endometrial elevation of LIF in junction with reductions in claudin 4 is further shown to correlate with a higher probability of pregnancy in IVF^44^. Our finding that PCX inhibits LIF but increases junction protein claudin, provides important molecular insights into why PCX negatively influences endometrial receptivity. This notion is further supported by the observation that PCX likewise suppresses other receptivity promoting factors such as CSF1, ERBB4, FGF2, TGFB1, and MMP14^40–45^. Conversely, PCX enhances the expression of WNT7A and LEFTY2, which are shown to be associated with implantation failure^46,47^. These results thus suggest that PCX promotes an anti-implantation epithelium by multiple means.

Our results are consistent with the principle that human endometrial epithelium is intrinsically non-receptive for embryo implantation, and that PCX must be down-regulated to open up the window of implantation. However, down-regulation of PCX throughout the endometrial tissue may have detrimental consequences because it would likely compromise the barrier functions of numerous glands as well as blood vessels. Instead, the endometrium selectively down-regulates PCX only in the luminal epithelial cells at receptivity^20^. Such a local PCX down-regulation only in cells that would directly interact with an implanting embryo, would selectively switch the endometrial surface to an implantation-permitting state but maintain the homeostasis of the entire organ. These findings and interpretations add important knowledge to the understanding of endometrial biology. These results also have important implications in the development of innovative approaches to optimize endometrial receptivity to improve IVF outcomes.

This study has some limitations. It would be ideal to use primary epithelial cells, however, it is impractical as normal endometrial tissues are scarcely available and epithelial cells isolated from these tissues are of low yield and cannot be maintain in culture for long. As such, Ishikawa cell line which is a commonly used receptive human endometrial epithelial cell line was utilized in this study. Overexpression of PCX in Ishikawa cells converted them into a non-receptive state, thus no other hormonal treatment was employed.

In summary, we discovered that PCX negatively regulates endometrial receptivity through several potential pathways, such as acting as an anti-adhesin and epithelial sealant and enforcing epithelial barrier functions, which inevitably prevent embryos from attaching or traversing the epithelial cell layer as demonstrated previously^20,21^. Moreover, PCX enhances various factors that are anti-implantation but dampens those that are pro-implantation. Collectively, PCX promotes the formation of an impermeable epithelium with a cellular profile that is inhibitory to embryo implantation. These results also have wider implications in the understanding of PCX in other epithelial cells outside the endometrium.

## Materials and methods

### Culture of control and PCX-overexpressing Ishikawa cells

Control and PCX-overexpressing (PCX-OE) Ishikawa cells, previously established in our laboratory^20^, were cultured in complete medium containing MEM (Thermo Fisher Scientific, USA) supplemented with 10% v/v FBS (Bovogen Biologicals, Australia), 1% v/v antibiotic–antimycotic, 1% v/v L-glutamine (Thermo Fisher Scientific) and 2% v/v G418 (Sigma Aldrich, USA). To generate these cells, expression construct of human PCX and empty pCMV6 vector (control plasmid) purchased from Origene (Rockville, USA) were transfected into Ishikawa cells with lipofectamine transfection reagent (Life technologies, USA). Final colonies were confirmed by real-time RT-PCR, western blot and immunocytochemistry analysis of PCX expression^20^.

### RNAseq analysis of control and PCX-overexpressing Ishikawa cells

Control and PCX-OE Ishikawa cells were cultured overnight in a 6-well plate in complete medium as mentioned above. The following day, cells were washed with PBS, total RNA was isolated using the RNeasy Mini Kit (Qiagen, Germany) and treated with TURBO DNA-free kit (Thermo Fisher Scientific).

RNAseq library preparation and sequencing was carried out at the University of Adelaide. Total RNA was converted to strand specific Illumina compatible sequencing libraries using the NEXTflex Rapid Directional mRNASeq library kit from BIOO Scientific (Austin, Texas) as per the manufacturer’s instructions (v14.10). Total RNA (400 ng) was polyA-selected and the mRNA fragmented prior to reverse transcription and second strand cDNA synthesis using dUTP. The resultant cDNA is polyadenylated before the ligation of Illumina-compatible barcoded sequencing adapters. The cDNA libraries were treated with UDG to degrade the second strand and PCR amplified for 14 cycles prior to assessment by Agilent Bioanalyzer for quality and Qubit fluorescence assay for amount. Sequencing pools were generated by mixing equimolar amounts of compatible sample libraries based on the Qubit measurements. Sequencing of the library pools was done with an Illumina Nextseq 500 using single read 75 bp v2 sequencing chemistry.

To identify differentially expressed genes, the raw 75 bp single-end FASTQ reads were first assessed for quality using FastQC^51^ and results aggregated using R/Bioconductor package *ngsReports*^52^. Reads were then trimmed for sequence adapters using *AdapterRemoval*^53^ and aligned to the human genome GRCh37 using the RNAseq alignment algorithm *STAR*^54^. The mapped sequence reads were then summarised to the GRCh37.p13 (NCBI:GCA_000001405.14 2013-09) gene intervals using *featureCounts*^55^, and the count table was transferred to the R statistical programming environment for expression analysis. Effect of sequence duplicates were also investigated using the function *MarkDuplicates* from the Picard tools package (http://broadinstitute.github.io/picard). Differential gene expression analysis was carried out with R/Bioconductor packages *edgeR*^56^ & limma^57^. The *limma voom*^58,59^ method, which uses log-counts and precision weight for each observation was employed to estimate variance and to apply a linear model.

### Real-time RT-PCR analysis of mRNA expression

Total RNA was extracted as described for RNAseq analysis, and total RNA (500 ng) was reverse transcribed using the Superscript III First-Strand Synthesis System (Thermo Fisher Scientific) as per manufacturer’s instructions. Semi-quantitative PCR was performed on the Applied Biosystems 7900HT fast real-time PCR system, using SYBR Green PCR Master Mix (Applied Biosystems, California, USA) and primers listed in supplementary Table S1.

### Immunofluorescence in control and PCX-overexpressing Ishikawa cells

Cells were grown on glass coverslips and fixed in either 4% (w/v) PFA (for analysis of E-cadherin, Wnt-7A, claudin 4 and ZO-1) or 100% methanol (for occludin). They were then blocked at room temperature (RT) with conditions optimized for each individual primary antibody: E-cadherin, 10% v/v horse serum (Sigma Aldrich) and 1% w/v BSA (Bovogen) in PBS for 1 h; Wnt7A, 10% v/v horse serum in PBS for 2 h; claudin 4, 10% v/v horse serum, 2% v/v human serum^60^, 0.1% w/v fish skin gelatin (Sigma Aldrich) and 0.1% v/v Triton X-100 (Sigma Aldrich) in PBS containing 0.2% v/v Tween20 (Sigma Aldrich) for 1 h; ZO-1, 1% BSA w/v in PBS for 2 h; occludin, 10% v/v goat serum (Sigma Aldrich), 2% v/v human serum, 0.1% w/v fish skin gelatin and 0.1% v/v Triton X-100 in PBS containing 0.2% v/v Tween20 for 1 h. Cells were then incubated overnight at 4 °C with primary antibodies for E-cadherin (2 µg/ml, ab1416, Abcam), Wnt7A (6 μg/ml, AF3008, R&D), claudin 4 (6 µg/ml, sc-376643, Santa Cruz), ZO-1 (10 µg/ml, 61-7300, Thermo Fisher Scientific), or occludin (1 µg/ml, 71-1500, Thermo Fisher Scientific). The cells were then washed 3 times with PBS and incubated at RT first with appropriate biotinylated secondary antibodies (Vector Laboratories) for 1 h, then with streptavidin conjugated Alexa Fluor 488 for 1 h. The nuclei were stained with DAPI for 5 min at RT (0.5 µg/ml in PBS, Sigma Aldrich), and fluorescence signal was visualized by confocal microscopy (Olympus Optical, Tokyo, Japan).

### Assessment of Ishikawa monolayer trans-epithelial electrical resistance and permeability

Permeable trans-well inserts (6.5 mm, 0.4 µm pore, Corning, USA) were pre-coated with 10 µg/ml fibronectin (BD Biosciences, USA). Control and PCX-OE Ishikawa cells were seeded (6 × 10^4^ cells per insert) on the upper chamber of the pre-coated insert in complete medium, and the lower chamber was filled with complete medium only. They were then incubated at 37 °C under 5% CO_2_ in a humidified incubator for 96 h, after which Trans-epithelial electrical resistance (TER) and monolayer permeability were assessed.

TER was measured using a Millipore MilliCell-Electrical Resistance System (Millipore, USA), while the cells were maintained at 37 °C using a warming plate. Prior to the measurement, the upper chamber was replenished with serum-free medium and the lower chamber with complete medium. Four TER readings (ohm per cm^2^) were taken from each well and readings from duplicate wells were averaged to obtain the raw measurement. The final TER value was obtained after subtracting background TER from wells containing no cells in the same experiment, and data were expressed as mean ± SD of three independent experiments.

Monolayer permeability was assessed by measuring the passage of fluorescein isothiocyanate (FITC)-conjugated dextran 40,000 from the upper to the lower chamber of the trans-well inserts. After the 96 h of culture, the bottom chamber was replenished with fresh complete medium and the upper chamber with fresh medium containing FITC-dextran (1 mg/ml, Sigma Aldrich), and the cells were incubated at 37 °C for 2 h. Media were then collected from the bottom chamber, diluted 1:5 in PBS, and fluorescence was measured at 485/535 nm (ClarioStar, BMG LabTech, Victoria, Australia). The final value was obtained after subtracting background fluorescence (PBS only), and data were expressed as mean ± SD of five independent experiments.

### Statistical analysis

R/Bioconductor packages *edgeR* was used for differential gene expression analysis. Other statistical analyses used GraphPad Prism version 9.01 (GraphPad Software, San Diego, CA) and unpaired t-test. Data were expressed as mean ± SD. Significance was defined as *P < 0.05; **P ≤ 0.005; ***P ≤ 0.0005, and ****P ≤ 0.0001.

## Supplementary Information

Supplementary Information.

## Data Availability

All data associated with this study are present in the paper or in the supplementary materials. All raw RNAseq data was submitted to the Gene Expression Omnibus (GEO) data repository with accession number GSE148274.

## References

[1] Ashary N, Tiwari A, Modi D (2018). Embryo implantation: War in times of love. Endocrinology.

[2] Evans J (2016). Fertile ground: Human endometrial programming and lessons in health and disease. Nat. Rev. Endocrinol..

[3] Nie G, Dimitriadis E, Kovacs G, Salamonsen LA (2019). How to prepare the endometrium to maximize implantation rates and IVF success.

[4] Salamonsen LA, Nie G, Hannan N, Dimitriadis E (2009). Society for Reproductive Biology Founders’ Lecture 2009. Preparing fertile soil: the importance of endometrial receptivity. Reprod. Fertil. Dev..

[5] Sharkey AM, Macklon NS (2013). The science of implantation emerges blinking into the light. Reprod. Biomed. Online.

[6] Dyer S (2016). International committee for monitoring assisted reproductive technologies world report: Assisted reproductive technology 2008, 2009 and 2010. Hum. Reprod..

[7] Chambers GM (2016). Population trends and live birth rates associated with common ART treatment strategies. Hum. Reprod..

[8] Fauser BCJM (2019). Towards the global coverage of a unified registry of IVF outcomes. Reprod. Biomed. Online.

[9] Revel A (2012). Defective endometrial receptivity. Fertil. Steril..

[10] Casper RF, Yanushpolsky EH (2016). Optimal endometrial preparation for frozen embryo transfer cycles: Window of implantation and progesterone support. Fertil. Steril..

[11] Kliman HJ, Frankfurter D (2019). Clinical approach to recurrent implantation failure: Evidence-based evaluation of the endometrium. Fertil. Steril..

[12] Bischof P, Campana A (1996). A model for implantation of the human blastocyst and early placentation. Hum. Reprod. Update.

[13] Lee KY, DeMayo FJ (2004). Animal models of implantation. Reprod.

[14] James JL, Carter AM, Chamley LW (2012). Human placentation from nidation to 5 weeks of gestation. Part I: What do we know about formative placental development following implantation?. Placenta.

[15] Aplin JD, Ruane PT (2017). Embryo–epithelium interactions during implantation at a glance. J. Cell Sci..

[16] Messaoudi S (2019). 15 years of transcriptomic analysis on endometrial receptivity: What have we learnt?. Fertil. Res. Pract..

[17] Lessey BA (2011). Assessment of endometrial receptivity. Fertil. Steril..

[18] Achache H, Revel A (2006). Endometrial receptivity markers, the journey to successful embryo implantation. Hum. Reprod. Update.

[19] von Grothusen C, Lalitkumar S, Rao Boggavarapu N, Gemzell-Danielsson K, Lalitkumar PG (2014). Recent advances in understanding endometrial receptivity: Molecular basis and clinical applications. Am. J. Reprod. Immunol..

[20] Paule SG (2021). Podocalyxin is a key negative regulator of human endometrial epithelial receptivity for embryo implantation. Hum. Reprod..

[21] Heng S (2021). Podocalyxin inhibits human embryo implantation in vitro and luminal podocalyxin in putative receptive endometrium is associated with implantation failure in fertility treatment. Fertil. Steril..

[22] Nielsen JS, McNagny KM (2008). Novel functions of the CD34 family. J. Cell Sci..

[23] Chen Q, Wang Y, Li Y, Zhao M, Nie G (2017). Serum podocalyxin is significantly increased in early-onset preeclampsia and may represent a novel marker of maternal endothelial cell dysfunction. J. Hypertens..

[24] Nielsen JS (2007). The CD34-related molecule podocalyxin is a potent inducer of microvillus formation. PLoS ONE.

[25] Nielsen JS, McNagny KM (2009). The role of podocalyxin in health and disease. J. Am. Soc. Nephrol..

[26] Shenolikar S, Voltz JW, Cunningham R, Weinman EJ (2004). Regulation of ion transport by the NHERF family of PDZ proteins. Physiology (Bethesda).

[27] Voltz JW, Weinman EJ, Shenolikar S (2001). Expanding the role of NHERF, a PDZ-domain containing protein adapter, to growth regulation. Oncogene.

[28] Weinman EJ (2001). New functions for the NHERF family of proteins. J. Clin. Invest..

[29] Orlando RA (2001). The glomerular epithelial cell anti-adhesin podocalyxin associates with the actin cytoskeleton through interactions with ezrin. J. Am. Soc. Nephrol..

[30] Schmieder S, Nagai M, Orlando RA, Takeda T, Farquhar MG (2004). Podocalyxin activates RhoA and induces actin reorganization through NHERF1 and Ezrin in MDCK cells. J. Am. Soc. Nephrol..

[31] Cait J (2019). Podocalyxin is required for maintaining blood-brain barrier function during acute inflammation. Proc. Natl. Acad. Sci. U.S.A..

[32] Alowayed N, Salker MS, Zeng N, Singh Y, Lang F (2016). LEFTY2 controls migration of human endometrial cancer cells via focal adhesion kinase activity (FAK) and miRNA-200a. Cell Physiol. Biochem..

[33] Cheng CW, Smith SK, Charnock-Jones DS (2008). Transcript profile and localization of Wnt signaling-related molecules in human endometrium. Fertil. Steril..

[34] Dominguez F, Yanez-Mo M, Sanchez-Madrid F, Simon C (2005). Embryonic implantation and leukocyte transendothelial migration: Different processes with similar players?. FASEB J..

[35] Flamini MI, Sanchez AM, Genazzani AR, Simoncini T (2011). Estrogen regulates endometrial cell cytoskeletal remodeling and motility via focal adhesion kinase. Fertil. Steril..

[36] Iwahashi M, Muragaki Y, Ooshima A, Yamoto M, Nakano R (1996). Alterations in distribution and composition of the extracellular matrix during decidualization of the human endometrium. J. Reprod. Fertil..

[37] Kusama K (2015). Regulatory Action Of Calcium Ion On Cyclic AMP-enhanced expression of implantation-related factors in human endometrial cells. PLoS ONE.

[38] Mohamed SA, Atta IS, Rowan BG, Desouki MM (2014). ERalpha and ERK1/2 MAP kinase expression in microdissected stromal and epithelial endometrial cells. J. Egypt Natl. Canc. Inst..

[39] Guzeloglu Kayisli O, Kayisli UA, Luleci G, Arici A (2004). In vivo and in vitro regulation of Akt activation in human endometrial cells is estrogen dependent. Biol. Reprod..

[40] Aghajanova L, Bjuresten K, Altmae S, Landgren BM, Stavreus-Evers A (2008). HB-EGF but not amphiregulin or their receptors HER1 and HER4 is altered in endometrium of women with unexplained infertility. Reprod. Sci..

[41] Guo F (2018). Decreased PECAM1-mediated TGF-beta1 expression in the mid-secretory endometrium in women with recurrent implantation failure. Hum. Reprod..

[42] Paiva P (2011). Human chorionic gonadotrophin regulates FGF2 and other cytokines produced by human endometrial epithelial cells, providing a mechanism for enhancing endometrial receptivity. Hum. Reprod..

[43] Robertson SA, Chin PY, Femia JG, Brown HM (2018). Embryotoxic cytokines-potential roles in embryo loss and fetal programming. J. Reprod. Immunol..

[44] Serafini PC (2009). Endometrial claudin-4 and leukemia inhibitory factor are associated with assisted reproduction outcome. Reprod. Biol. Endocrinol..

[45] Thathiah A, Carson DD (2004). MT1-MMP mediates MUC1 shedding independent of TACE/ADAM17. Biochem. J..

[46] Fan X (2012). Dynamic regulation of Wnt7a expression in the primate endometrium: Implications for postmenstrual regeneration and secretory transformation. Endocrinology.

[47] Salker MS (2018). LEFTY2 inhibits endometrial receptivity by downregulating Orai1 expression and store-operated Ca(2+) entry. J. Mol. Med. (Berl).

[48] Cullinan EB (1996). Leukemia inhibitory factor (LIF) and LIF receptor expression in human endometrium suggests a potential autocrine/paracrine function in regulating embryo implantation. Proc. Natl. Acad. Sci. U.S.A..

[49] Hambartsoumian E (1998). Endometrial leukemia inhibitory factor (LIF) as a possible cause of unexplained infertility and multiple failures of implantation. Am. J. Reprod. Immunol..

[50] Mariee N, Li TC, Laird SM (2012). Expression of leukaemia inhibitory factor and interleukin 15 in endometrium of women with recurrent implantation failure after IVF; correlation with the number of endometrial natural killer cells. Hum. Reprod..

[51] Andrews S. *FastQC: a quality control tool for high throughput sequence data. Available online at: *http://www.bioinformatics.babraham.ac.uk/projects/fastqc <http://www.bioinformatics.babraham.ac.uk/projects/fastqc > (2010).

[52] Ward CM, Thu-Hien T, Pederson SM (2019). ngsReports: A bioconductor package for managing FastQC reports and other NGS related log files. Bioinformatics.

[53] Schubert M, Lindgreen S, Orlando L (2016). AdapterRemoval v2: Rapid adapter trimming, identification, and read merging. BMC Res. Notes.

[54] Dobin A (2013). STAR: Ultrafast universal RNA-seq aligner. Bioinformatics.

[55] Liao Y, Smyth GK, Shi W (2014). featureCounts: An efficient general purpose program for assigning sequence reads to genomic features. Bioinformatics.

[56] Robinson MD, McCarthy DJ, Smyth GK (2010). edgeR: A Bioconductor package for differential expression analysis of digital gene expression data. Bioinformatics.

[57] Ritchie ME (2015). limma powers differential expression analyses for RNA-sequencing and microarray studies. Nucleic Acids Res..

[58] Law CW, Chen Y, Shi W, Smyth GK (2014). voom: Precision weights unlock linear model analysis tools for RNA-seq read counts. Genome Biol..

[59] Liu R (2015). Why weight? Modelling sample and observational level variability improves power in RNA-seq analyses. Nucleic Acids Res..

[60] Li Y (2011). Placental HtrA3 is regulated by oxygen tension and serum levels are altered during early pregnancy in women destined to develop preeclampsia. J. Clin. Endocrinol. Metab..

