# Supplementary material for: Podocalyxin promotes an impermeable epithelium and inhibits pro-implantation factors to negatively regulate endometrial receptivity
Source: Sci Rep. 2021 Dec 14;11:24016. doi: 10.1038/s41598-021-03425-2 (PMC8671585; doi:10.1038/s41598-021-03425-2)
Supplement: Supplementary file 1 — Supplementary Information. [file 41598_2021_3425_MOESM1_ESM.pdf]

Figure S1: Multi-dimensional Scaling (MDS) plot displaying unsupervised clustering of gene expression profiles of control (CON) and PCX-overexpressing (PCX-OE) Ishikawa cells.

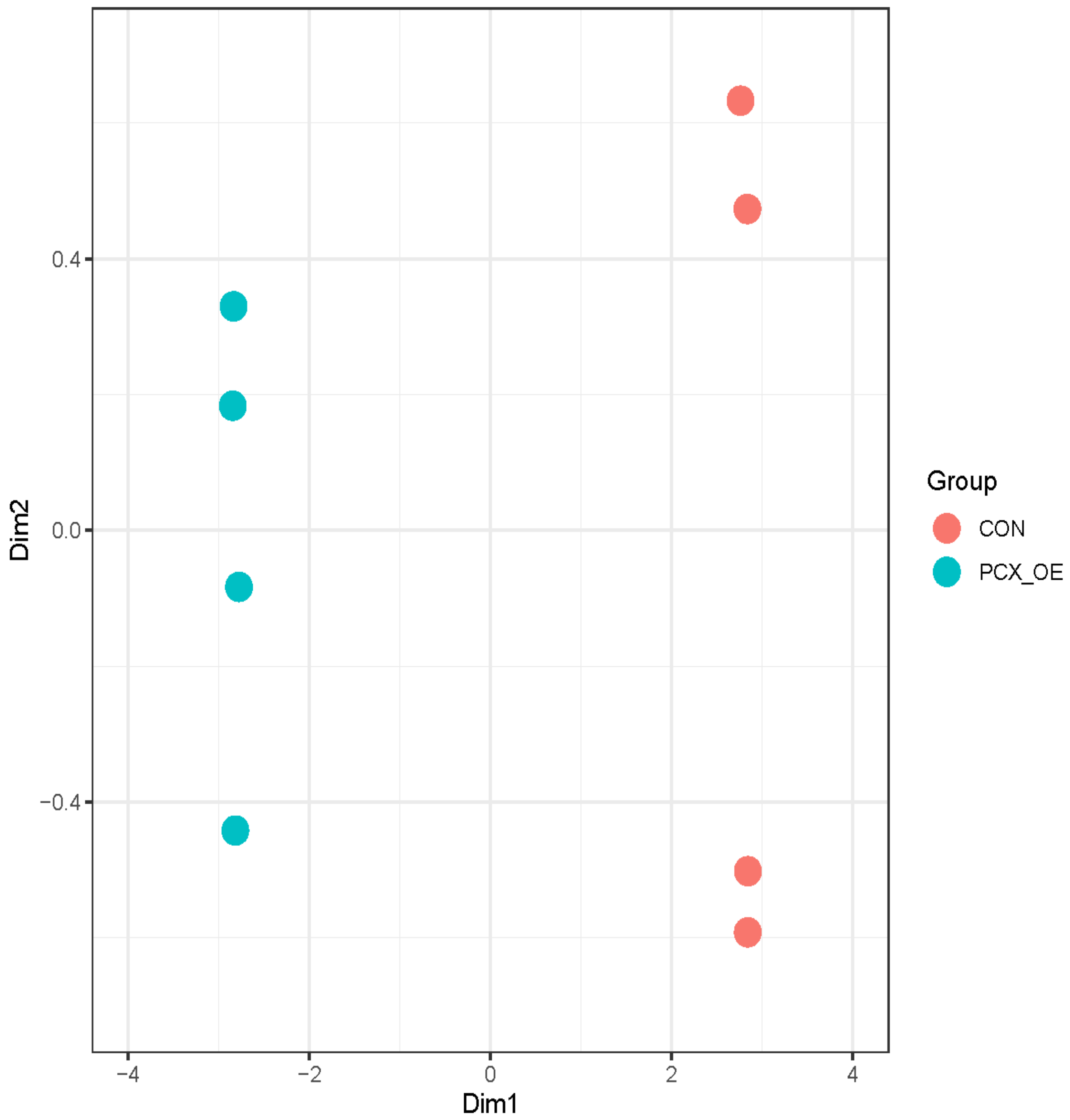

Table S1: Primer sequences used for RT-PCR analysis.

Gene	Primer sequence (5'→3')	
	Forward	Reverse
CDH1	GAAGGTGACAGAGCCTCTGGAT	GATCGGTTACCGTGATCAAAATC
TJP1	GGGAACAACATACAGTGACGC	CCCCACTCTGAAAATGAGGA
CLDN4	CCCCGAGAGAGAGTGCCCTG	AGCGTCCACGGGAGTTGAGGA
OCLN	CTCTCTCAGCCAGCCTACTC	GTTCCATAGCCTCTGTCCCA
WNT7A	TGCCCGGACTCTCATGAAC	GTGTGGTCCAGCACGTCTTG
LEFTY2	CTGGACCTCAGGGACTATGG	TCAATGTACATCTCCTGGCG
LIF	TGCCAATGCCCTCTTTATTC	GTTGACAGCCCAGCTTCTTC
CSF1	TAGCCACATGATTGGGAGTGGA	CTCAAATGTAATTTGGCACGAGGTC
ERBB4	GATGATCGTATGAAGCTTCCCA	CGGTATACAAACTGGTTCCTATTC
FGF2	CGGATGGGGGTAGTGAGCA	ATCTTGAGGTGGAAGGGTCT
TGFB1	CAACAATTCCTGGCGATACCT	GCTAAGGCGAAAGCCCTCAAT
MMP14	GCAGAAGTTTTACGGCTTGCA	TCGAACATTGGCCTTGATCTC
YWHAZ	CCGCCAGGACAAACCAGTAT	ACTTTTGGTACATTGTGGCTTCAA
